# Diagnosing and managing patients with heart failure with preserved ejection fraction: a consensus survey

**DOI:** 10.1136/bmjopen-2024-092993

**Published:** 2024-12-20

**Authors:** Rosalynn Austin, Eva Khair, Thomas Blakeman, Muhammad Zakir Hossain, Emma Sowden, Carolyn Chew-Graham, Faye Forsyth, Christi Deaton, for the Optimise HFpEF Investigators, Mollika Chakravorty

**Affiliations:** 1 Department of Public Health, University of Stavanger, Stavanger, Norway; 2 Cardiology Research, Portsmouth Hospitals University NHS Trust, Portsmouth, UK; 3 Department of Public Health and Primary Care, University of Cambridge School of Clinical Medicine, Cambridge, UK; 4 Anglia Ruskin University School of Clinical Medicine, Chelmsford, UK; 5 University of Manchester, Manchester, UK; 6 Birmingham City University, Birmingham, UK; 7 School of Medicine, Keele University, Newcastle-under-Lyme, UK; 8 Department of Public Health and Primary Care, KU Leuven, Leuven, Belgium

**Keywords:** Primary Care, Health Services, Heart failure

## Abstract

**Abstract:**

**Aim:**

As heart failure (HF) with preserved ejection fraction (HFpEF) prevalence increases, it remains frequently underdiagnosed and poorly managed. Recent positive pharmacological trials have increased interest in HFpEF but challenges of diagnosis and management remain. The survey aim was to examine consensus between primary and secondary care providers regarding HFpEF diagnosis and management.

**Methods:**

As part of a larger programme of work, survey questions were developed in an online format and piloted with healthcare providers (HCPs). The survey link was distributed via professional networks and social media. Analysis included frequencies of responses, comparison by main professional groups and thematic analysis free-text responses. A virtual workshop of HCPs was conducted to discuss and refine survey findings.

**Results:**

HCPs (n=66) across the UK participated: 19 general practitioners (GPs), 20 HF specialist nurses (HFSN), 17 cardiologists and 10 others. Consensus was high (92%) that diagnosing the type of HF was very important and most favoured inclusion of HFpEF in Quality Outcome Framework indicators. No clear consensus was reached that ongoing management should be in primary care (47.5% of GPs, 35% of HFSN and 31.3% of cardiologists ‘somewhat agreed’). Opinions differed between GPs (52.3)% and specialists (HFSN 80% and cardiologists 81.3%) for practice nurses to be upskilled and assume HFpEF management. No HCPs reported any level of disagreement for HFSN management of HFpEF. Free-text comments highlighted resource barriers to HFpEF diagnosis and management and confirmed the need to develop better HFpEF services.

**Conclusions:**

Consensus was reached regarding importance of diagnosing HFpEF, but agreement on methods and responsibilities for diagnosis and management varied. Free-text comments identified HCPs concerns related to overwhelmed primary and secondary care services and lack of sufficient resources to meet existing patient demands. Creation of collaborative care pathways is needed to support the increasing number of older patients with HFpEF.

**Trial registration number:**

ClinicalTrials. gov (reference number: NCT03617848).

**STRENGTHS AND LIMITATIONS OF THIS STUDY**

- Survey collected the opinions and thoughts of multiple types of healthcare providers across both secondary and primary care in the UK.
- Findings of the survey were confirmed by an independent workshop of healthcare providers and members of the public.
- Answers in free-text boxes were analysed and provided context and depth to the survey responses.
- Survey was conducted prior to the research which demonstrated the usefulness of medications in this population.
- Despite widespread distribution, the number of healthcare professionals who completed the survey was limited; however, a degree of geographical representation across the UK was achieved (majority from England).

## Introduction

 Heart failure (HF) with preserved ejection fraction (HFpEF) is a heterogenous clinical syndrome which accounts for at least half of total HF cases.^1^,^3^ The prevalence of HFpEF is increasing commensurate with an ageing population living with comorbid conditions that drive the development of HFpEF.^2 3^ Despite its prevalence, HFpEF has remained underidentified^4^ and often poorly managed.^5 6^ In part, this reflects greater challenges in diagnosis and less clearly defined treatment pathways for patients with HFpEF, compared with those with heart failure with reduced ejection fraction (HFrEF). Protracted diagnostic pathways, scepticism regarding the condition, therapeutic nihilism and lack of access to specialist services have been a common experience for patients with HFpEF.^5 6^

Previously, the mainstay of HFpEF management was management of symptoms and treatment of underlying conditions. Recent pharmacological trials of sodium glucose co-transporter-2 inhibitors (SGLT2i) and glucagon-like peptide receptor agonists (GLP-1) have shown improved outcomes in patients with HFpEF. SGLT2i reduced the composite endpoint of HF hospitalisations and cardiovascular mortality in the Empagliflozin in Heart Failure with a Preserved Ejection Fraction (EMPEROR-Preserved) and Dapagliflozin in Heart Failure with Mildly Re duced or Preserved Ejection Fraction (DELIVER) trials.^7 8^ SGLT2i are approved for use in HF regardless of ejection fraction.^9 10^ More recently, the Semaglutide in Patients with Heart Failure with Preserved Ejection Fraction and Obesity (STEP-HF) trial reported that treatment with the GLP-1 agonist, semaglutide, for patients with HFpEF and obesity, led to greater weight loss, reduction in an inflammatory marker, improved symptoms and physical function compared with placebo.^11^ These studies provide an impetus for diagnosis and treatment of patients with HFpEF, but the ability of the healthcare system to provide timely diagnostic and management services remains a concern.^12^

An analysis of over 75 000 patients with HF in Sweden found that less than half of patients with HFpEF were referred for specialist care compared with 73% of those with HFrEF.^13^ A recent survey of HF services in the UK found that community HF teams reviewed 100% of patients with HFrEF, and 57% of those with HFpEF. In-hospital HF teams reviewed 96% of primary HFrEF admissions and 69% of primary HFpEF admissions (J Masters, unpublished data, 2025). Although National Institute for Health and Clinical Excellence (NICE) guidelines for HF call for multidisciplinary team management that includes primary care, a high level of collaboration between specialist services and primary care is seldom realised.^6 14^

Despite the responsibility of HFpEF management mainly residing with primary care clinicians, the quality outcomes framework (QOF) does not specify HF type. The QOF is a ‘voluntary annual reward and incentive programme for all general practitioner (GP) practices in England’, detailing practice achievement results.^15^ It rewards good practice based on achievement of indicators across five domains, including management of clinical conditions such as HF. QOF indicators for HF are to maintain a register of patients with HF, have HF diagnosis confirmed by echocardiogram or specialist review, ensure that patients with HFrEF are taking ACE inhibitors (ACEI) and beta blockers (BB) unless contraindicated, and that patients on the HF register have been called in for a review in the last 12 months.^16^

Given the increasing prevalence of HFpEF, and challenges seen in its recognition, diagnosis and management, we undertook a study to determine the degree of consensus between primary and secondary care healthcare providers (HCPs) regarding HFpEF diagnosis and management.

The survey was conducted prior to the positive pharmacological trials, but the issues of ensuring robust services for diagnosis and management of complex groups of patients remain relevant.

## Methods

A cross-sectional survey of HCPs across primary care and specialist services was conducted. The survey was a component of the Optimising Management of Heart Failure with Preserved Ejection Fraction in Primary Care (Optimise HFpEF) programme of research.^17^ Survey questions were developed for an online format by Optimise HFpEF researchers using the Qualtrics platform. Questions covered areas identified as problematic from previous findings of the Optimise HFpEF research^6 18 19^ and in relevant literature. Survey questions were iteratively developed by the research group and the initial survey was piloted with five HCPs leading to refinement. The final survey included 14 questions, with two being free text. The survey is available in the online supplemental material 1.

Sampling was a combination of virtual snowballing and self-selection. The survey link was distributed to HCPs via professional networks and colleagues of investigators (including HCPs who had taken part in Optimise HFpEF studies) who were asked to further cascade to practices and other HCPs. The link was advertised via social media accounts, primarily Twitter (now X). Although impossible to determine, potentially hundreds of HCPs could have received the survey link. Using snowballing technique participants were invited across the four nations of the UK. Participation was through self-referral and anonymous, with exclusions limited to incomplete surveys (n=3). Survey data were collected between December 2020 and March 2021.

The online Qualtrics platform enabled quantitative, categorical and text data collection. Descriptive analysis was performed on (questions 1–4, 6–13) to determine frequencies of responses in MS Excel.^20^ Responses were grouped as positive (eg, slightly agree, agree, strongly agree), negative (eg, slightly disagree, disagree, strongly disagree) and neutral (eg, neither agree nor disagree) to facilitate the assessment of consensus. Consensus level was set at 2/3 (67%).^21^ Answers to the survey questions were compared in the three most prevalent HCP types: GPs, heart failure specialist nurses (HFSN) and cardiologist. Two questions (5 and 14) asked respondents to provide detailed responses in a free-text box. Text data were organised using Nvivo^22^ and analysed thematically by three of the authors (RA, EK and CD). A pragmatic qualitative approach was used to both identify, characterise and explain the data observed in the free-text boxes. These data were integrated using a narrative approach with the survey results.^23^

### Public and HCP involvement

A virtual workshop with HCPs and public not involved in the survey was held in September 2021 to discuss the findings from the consensus survey and next steps in practice. The group was led in a discussion of a systems approach to healthcare^24^ within the context of survey findings. Interactive software was used to enable participants to provide input using virtual post-it notes to specific categories related to identification of problems and potential improvements in practice. Post-it notes were discussed in the workshop and collated at the end of the meeting.

Participant information and electronic consent were at the start of the survey and consent was required to proceed to the survey questions.

## Results

HCPs (n=66) completed the survey across the UK. Respondents came 31% from the East of England region, 43% from seven other regions in England, 12% from Northern Ireland, 7.7% from Wales,1.5% from Scotland and 9.7% were unknown. Respondents self-selected an age range (10-year groupings) from 21 to 30 up to 61–70. The majority, 66%, chose the age range between 41 and 60 years old. Respondents were primarily female (53%) and two identified as non-binary. Clinical professional group representations were as follows: 19 GPs (GP (n=14), GP with special interest in cardiology (n=2), trainee GP (n=2) and GP registrar (n=1)), 20 HFSNs, 16 cardiologists and nine other HCPs (practice nurse (n=1), student nurse (n=1), research nurse (n=1), researchers (n=3), allied health professionals (n=1), other medical specialist (n=1), junior doctor (n=1)), and two unknown. Due to insufficient responses in the *other* HCP category, survey responses were only compared among GPs, HFSP and cardiologists, reducing the total sample to 55 included in the results; missing data were labelled as unknown.

The survey results were divided into two main points for discussion: the diagnosis of HFpEF and the management of HFpEF.

### Diagnosis of HFpEF

**Figure 1:**
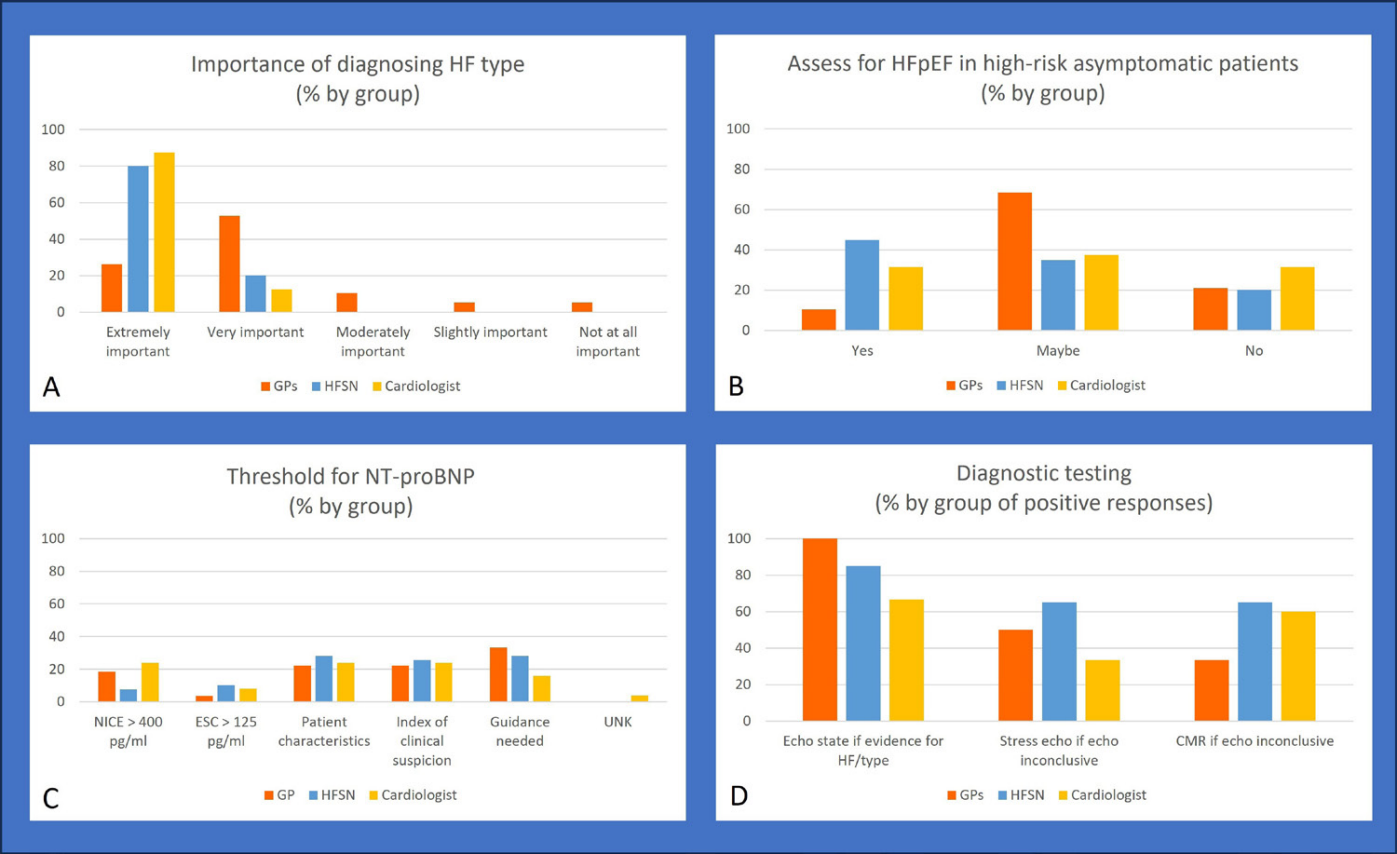
Survey responses from questions around diagnosis of HFpEF. (A) Importance of diagnosing HF type, (B) asses for HFpEF in high-risk asymptomatic patients, (**C**) threshold for N-terminal pro B-type natriuretic peptide (NT-pro BNP), and (D) diagnostic testing.CMR, Cardiovascularc Magnetic Resonance; ESC, European Society of Cardiology; GP, general practitioner; HF, heart failure; HFpEF, heart failure with preserved ejection fraction; HFSN, heart failure specialist nurse; UNK, unknown.

Consensus was reached by aggregating all positive responses across all HCPs that diagnosing HF type was important (GPs 78%, HFSN 100%, cardiologist 100%). Specialist HCPs (cardiologists: 87.5%, n=14 and HFSN: 80%, n=16) respondents all reported that diagnosing HF type was extremely important. A lower proportion of GPs (26.3%, n=5) in comparison considered it extremely important and 52.6% (n=10) thought it very important. GPs were the only HCP type to consider HF diagnosis not at all important (5.3%, n=1), slightly important (5.3%, n=1) and moderately important (10.5%, n=2). See figure 1A for details.

Question 10 provided HCPs the opportunity to choose a fixed response as to *why or why not* identifying HFpEF was useful. HCPs (41.8%, n=23) thought it useful to make a definite diagnosis of type of HF without specifying a particular reason. Just under half (n=23) of the sample chose that identifying HFpEF was an opportunity to focus on comorbidities and lifestyle factors. Four per cent (n=2) of the respondents did not think it useful as treatment would not differ, and 9% (n=5) thought it provided an opportunity to deprescribe medications.

Adding to the reasons for the importance of identifying HF type came from responses from the free-text question 15. Respondents reported that diagnosis would facilitate both tailoring treatments and service pathways together with collecting epidemiological data for HF phenotypes (exemplar quotes in table 1).

**Table 1:** Reasons for identifying type of HF, exemplar quotes

HCP type, location	Exemplar data extracts
Tailoring treatments and services	
Cardiologist, England	When it comes to HFpEF we need not only to diagnose the condition correctly but also the various sub-phenotypes—as in due course it is likely that some of these sub-phenotypes will benefit from pharmacotherapy.
GPwSI cardiology, England	Treatments are likely to be better in the near future, so more important to diagnose now than in the past.
HFSN, England	Patients with HFpEF are often prescribed standard HFrEF medications in the mistaken belief that the treatment is the same for any type of HF, not because they are indicated for other reasons, for example, Beta-blocker for heart rate control, ACE-inhibitor for hypertension. As this is generally an elderly population we need to rationalise/ take great care not to cause further problems such as falls due to inappropriate prescribing.
HFSN, England	Some services based on EF & excluded HFpEF. Enables you to design services that are inclusive and can be tailored.
Importance of epidemiological data	
HFSN, England	So we can audit exactly who has HFpEF vs HFrEF and maybe add in more resources for this cohort of patients. We believe 50% of patients have HFpEF and have little support in the community.
Cardiologist, NI	Important to have epidemiological info on incidence.

EFejection fractionGPwSIgeneral practitioner with special interestHCPhealthcare professionalHFheart failureHFpEFheart failure with preserved ejection fractionHFrEFheart failure with reduced ejection fractionHFSNheart failure specialist nurseNINorthern Ireland

While consensus was reached across HCP types (GPs 78.9%: n=15, HFSN 80%, n=16 and cardiologists 81.3%, n=13) to support the assessment of HFpEF among symptomatic patients, there was less agreement for assessing high-risk patients if asymptomatic (figure 1B). GPs were equivocal and replied ‘maybe’ (68%, n=13) where only 10.5% (n=2) agreed screening should be done. Specialists were also divided on whether high-risk asymptomatic patients should be screened (yes: HFSN 45%, n=9; cardiologists 31%, n=5; maybe: HFSN 35%, n=7; cardiologists 37.5%, n=6).

A key factor in diagnosis for HF and onward referral for further investigations is a patient’s natriuretic peptide (NP) value. Respondents were asked to either identify their preferred threshold for N-terminal pro B-type natriuretic peptide (NT-pro BNP), using different thresholds between ESC and NICE, and/or given the option to answer that thresholds should vary by patient characteristics, index of clinical suspicion, or whether better guidance was needed. Respondents were permitted to choose multiple responses to this question; a total of 91 responses were received. All HCP types chose more than one answer (n=22) (see figure 1C). No consensus was reached around the factors which should be considered for an NT-pro BNP threshold, with HCPs commonly choosing the choice that the NP value needs to be considered in conjunction with a patient’s characteristics (n=23 responses) and clinical index of suspicion (n=23 responses). All HCP types called for better guidance (GPs 47.4%, n=9, HFSNs 55%, n=11; cardiologists 25%, n=4).

Two respondents (in the free-text boxes) mentioned patient characteristics that would influence their interpretation of NP thresholds: heart rhythm (sinus or atrial fibrillation) and obesity.

“Do we have data to demonstrate how great is the exact impact of obesity on natriuretic peptides? […] I think it would help to disseminate the message that the NPs are probably elevated when there’s a degree of decompensation, and that adequate ‘treatment’ […] can normalise the values (so give a false negative). Also, it would help to know to what degree AF [atrial fibrillation] impacts on NPs [natriuretic pepetide]” GP, England

“Different cut [off] points should be used to diagnose HFpEF depending on whether patients are in sinus rhythm or atrial fibrillation. This must take into account that treatment with diuretics may reduce/normalise NTproBNP levels. Also, obese patients have lower NTproBNP levels.” Cardiologist, England

The use of echocardiogram results or additional tests (ie, stress echocardiogram and cardiac MRI (CMR)) to confirm a diagnosis of HFpEF elicited varying opinions. Consensus reached across all clinician types that echo reports need to state the presence of evidence of HF types. GPs unanimously agreed (somewhat agree to strongly agree) that echocardiogram reports should state if there was evidence of HF and the type of HF. The use of stress echocardiograms or CMR if the resting echocardiogram was inconclusive received less support by group with only specialists having similar levels of agreement (figure 1D). Some respondents (30%) were neutral (neither agreed nor disagreed) with the use of stress echocardiogram or CMR for diagnostic purposes.

Comments in free-text question regarding the use of additional testing for diagnosis acknowledged the heterogeneity of HFpEF presentation. Clinicians reported needing to carefully consider the appropriateness for a particular patient before requesting additional tests. Specialists (cardiologists and HFSN) argued that an echocardiogram does not diagnose HF, as HF is a clinical diagnosis, but stated the importance of measuring echocardiogram parameters required for HFpEF identification. CMR, while not routine, was thought useful by a cardiologist for determining underlying aetiology such as cardiomyopathies or amyloidosis.

### Quality and outcomes framework

**Figure 2:**
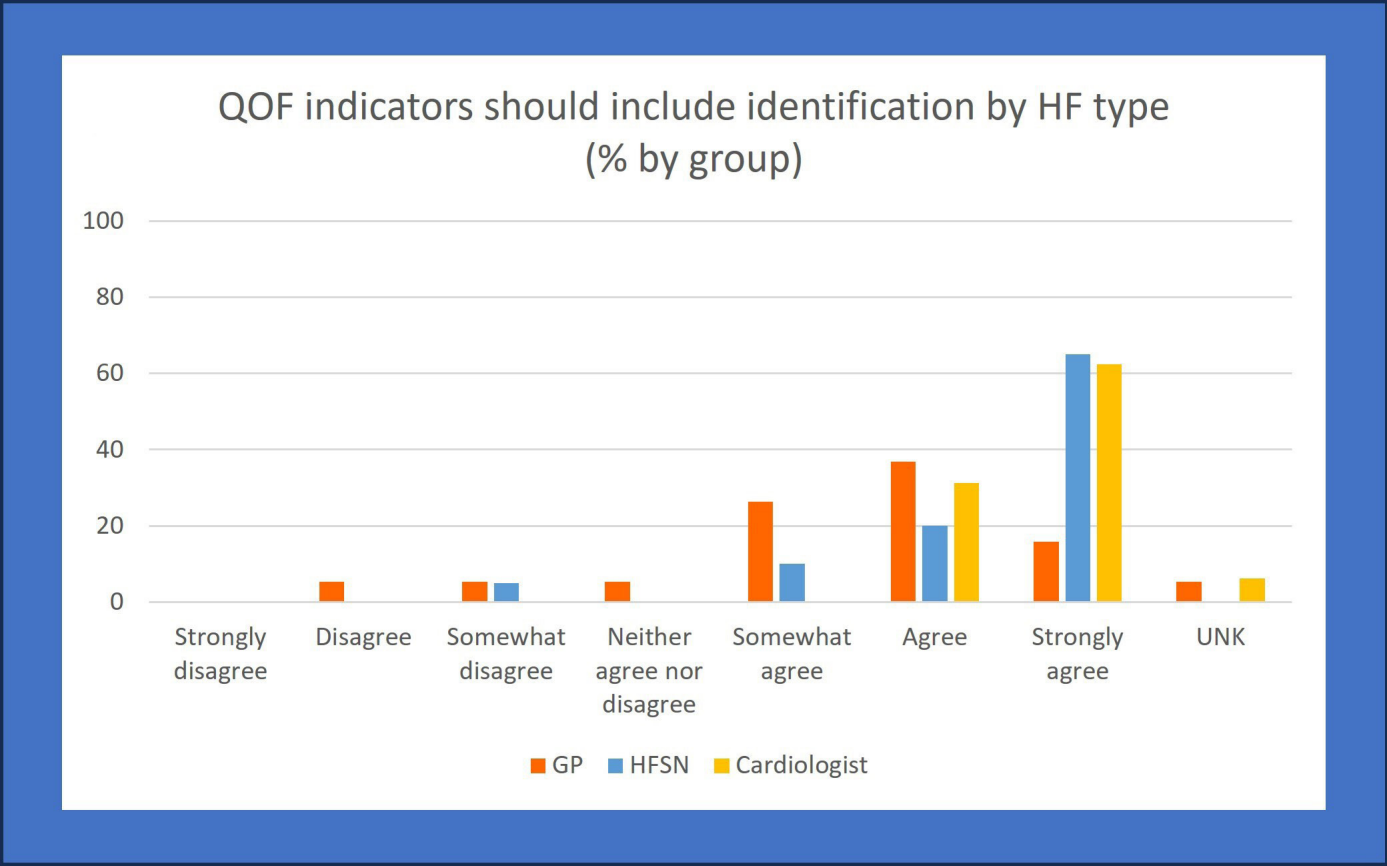
Survey responses from the question of whether QOF indicators should include HF type identification. GP, general practitioner; HF, heart failure; HFSN, heart failure specialist nurse; QOF, quality outcome frameworks; UNK, unknown.

Question 15 asked if the QOF within primary care should be changed to include an indicator for type of HF being documented. Consensus was reached across all clinicians. Most specialists responded as agree or strongly agree (cardiologists: 93.7%, n=15 and HFSN: 85%, n=17) to include HFpEF as a QOF indicator. While GPs reached the level of consensus (aggregated positive response), 11% (n=2) disagreed with the inclusion of HFpEF in the QOF (Figure 2)

Respondents could provide rationale for agreeing or disagreeing a change to QOF indicators for HF in a free-text box. Most respondents (n=49) provided details on their opinions regarding a change to the QOF indicators in primary care settings. Although some GPs and HFSNs responded negatively, the full-text responses demonstrated that these professionals instead had mixed opinions on the QOF including HFpEF type. Only a single GP was clearly against HFpEF type being included in the QOF, supported by their free-text comments. Most generalist and specialist clinicians expressed how they felt this change would improve patient identification, ensure treatment was adequate and improve services (exemplar quotes in table 2).

**Table 2:** Opinions regarding changes QOF indicators

HCP type	Exemplar data extracts, location
Positive opinions supporting change in QOF indicators	
GPs	(Yes to QOF) For clarity, more accurate record and evidence for prevalence. GP registrar, England
	Types of HF have very different treatment strategies so distinguishing them is important. QOF is a good way for “force” this and should not have huge impact in terms of workload as HF patient numbers are relatively low. England
HFSNs	Documenting the type of HF will help explain why patients with HFpEF aren’t on certain medications (BB, ACE I/ ARB/ ARNI). Northern Ireland
Cardiologists	Important so that optimisation of evidence-based treatment can be rewarded. Also encourages GPs to verify diagnoses better. England
	Will provide better population data re prevalence and also ensure best evidence-based care is provided. England
Mixed opinions of changes to QOF to included identification of HF type	
GPs	I do not feel I know enough about HFpEF to state if it should be included in QOF. England
	The diagnosis of HFpEF is sometimes tricky, needing specialist involvement (and even then the diagnosis can be nuanced) so hard to put upon GPs-unless better guidance becomes available. England
HFSNs	Treatment differs according to type of HF, so needs to be explicit. If GP unable to be explicit, suggests has not had adequate investigations/specialist review, so should prompt referral, as per NICE guidance. England
Cardiologists	Community heart failure specialist nurse teams not commissioned for HFpEF. England
Negative opinion of QOF to included identification of HF type	
GPs	For QoF coding the importance is on keeping a register of the condition and being able to recall patients and provide the defined care. The specific type of HF is relevant to the clinician on reviewing the notes/adjusting medication, it doesn’t need to be defined that specifically in QoF. England

BBbeta blockerGPgeneral practitionerHCPhealthcare professionalHFheart failureHFpEFheart failure with preserved ejection fractionHFSNheart failure specialist nurseQOFquality and outcomes framework

### Management of patients with HFpEF

**Figure 3:**
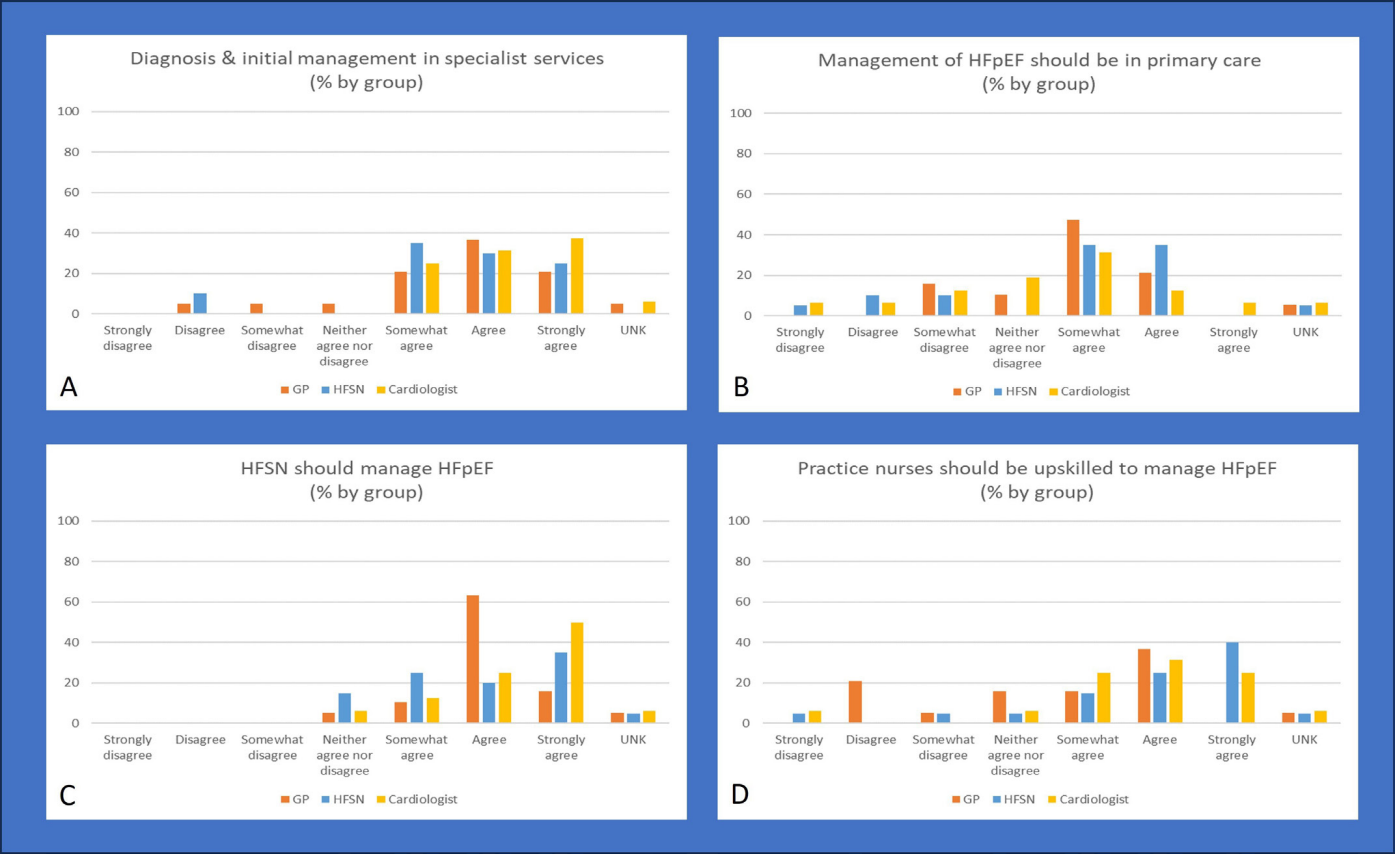
Survey responses from questions around management of HFpEF. (A) Diagnosis and initial management in specialist services. (B) Management of HFpEF should be in primary care. (C) HFSN should manage HFpEF. (D) Practice nurses should be upskilled to manage HFpEF. GP, general practitioner; HF, heart failure; HFpEF, heart failure with preserved ejection fraction; HFSN, heart failure specialist nurse; UNK, unknown.

Survey questions 10–13 asked about where and by whom patients with HFpEF should be managed. The first question on this topic assessed agreement to the statement “diagnosis and initial treatment plan for patients with HFpEF should happen in specialist services”. While consensus was not reached within individual HCP types, it was reached when the positive responses were aggregated for all clinical types (GPs 78.9%, n=15; HFSN 90%, n=18; cardiologists 93.8%, n=15). A few GPs (10.5%, n=2) strongly to somewhat disagreed with the statement (figure 3A).

The next questions asked respondents about their agreement that ongoing management of patients with HFpEF should be in primary care. No clear consensus was reached, although the largest category by all three groups was ‘somewhat agree’ with 47.5% (n=9) of GPs, 35% (n=7) of HFSN and 31.3% (n=5) of cardiologists choosing this response (figure 3B). Aggregated positive responses saw consensus reached by GPs (68.4%, n=13) and HFSN (70%, n=14), but not in cardiologists (50%, n=8).

Final survey questions probed the responsibilities of HFSN and practice nurses in managing patients with HFpEF. No respondents chose a negative response regarding HFSN management of patients with HFpEF, but agreement level varied by the groups (figure 3C). Aggregation of positive responses saw consensus reached across all HCP types (GPs 89.5%, n=17; HFSN 80%, n=16; cardiologists 87.5%, n=14).

Respondents had greater variation in their responses when it came to agreeing if upskilling practice nurses to assume the management of patients with HFpEF was appropriate (figure 3D). If positive responses were aggregated, then consensus was reached by HFSN (80%, n=16) and cardiologists (81.3%, n=13) but not by GPs (52.3%, n=10). Unlike responses to the responsibilities of HFSN being expanded to include HFpEF, when it came to considering the same for practice nurses there were negative responses. Important to note the voice of practice nurses was not in this analysis as despite widespread study distribution only one practice nurse responded. That individual was supportive of practice nurse management of HFpEF and did not agree to HSFN management of those with HFpEF.

Nuanced understanding of these responses was found in analysing free-text comments which asked respondents to share their thoughts on the management of those with HFpEF. Thematic analysis of those responses revealed that clinicians (both GPs and specialists) identified ways to improve HFpEF management and identified existing management barriers (table 3).

**Table 3:** Exemplar data extracts on management of those with HFpEF

	HCP type, location	Data extract
Management of HFpEF		
Complexity requires specialist	GP, England	Diagnosis and treatment should be made and initiated in secondary care. Specialist nurses can drive early stage as its protocol driven.
	HSFN,Northern Ireland	I strongly argue that patients with this condition require specialist and expert heart failure services.
	Cardiologist, Northern Ireland	Important to identify small subset of patients who have other treatable conditions, amyloid/constriction and therefore initial assessment by specialist is probably reasonable.
Call for integrated model of care	HFSN,England	We should use the skills of all the MDT in managing HFpEF and this will include the involvement of practice nurses who may be able to manage the more stable patients.
	HSFN,Wales	Might be a one off and then to PN [practice nurse] to manage or might be more needed based on specialist assessment.
	Cardiologist, Northern Ireland	Initial assessment by specialist is probably reasonable. Long term management could probably be in primary care.
Management needs	GP,England	Treatments are likely to be better in the near future, so more important to diagnose now than in the past.
	HFSN,Wales	I also think there needs to be a far more robust preventative medicines approach to care.
	HFSN,England	Non-pharmacological treatment options & plans.
	Cardiologist, Northern Ireland	There is nowhere near enough emphasis on prevention at present.
Barriers to HFpEF Management		
Limited treatments	GP,England	This is a challenging area, due to lack of therapeutic options and more research and support for patients is definitely needed.
	HFSN, England	I think everyone should understand HFpEF. People (nurses /doctors) are dabbling in the care without really evidence of treatment.
	HFSN, Northern Ireland	Limited evidence-based treatments, therefore management may not be optimal.
	Cardiologist, England	There is precious little guidance within this group.
Unclear diagnostic criteria	GP,England	Sometimes it appears to be a diagnosis of exclusion (normal echo, no other cause for SOB, + swollen ankles for example). It can feel unclear.
	HFSN,England	Stating the patient has ‘heart failure’ is a clinical diagnosis including symptoms—you can’t say somebody has HF from an echo alone.
	Cardiologist, England	Different cut points should be used to diagnose HFpEF depending on whether patients are in sinus rhythm or atrial fibrillation. This must take into account that treatment with diuretics may reduce/normalise NTproBNP levels. Also obese patients have lower NTproBNP levels.
Staff training and workloads	GP,England	I think this is an area GPs really struggle and understanding its importance and implications isn’t fully understood by all GPs.
	GP,England	In an ideal world it would be great for practice nurses to be upskilled to manage these patients(…)but many are struggling to cover the demands of all their roles currently and cannot keep expanding at present.
	HFSN,Northern Ireland	Upskill and more guidance on treatment is needed.
	HFSN,England	This will not be possible with existing resources—will need investment to provide a gold standard service.
	Cardiologist, Northern Ireland	Although I feel that it is important to recognise HFpEF and address risk factors, the health service cannot currently cope with demand for HFrEF recognition and treatment which has very definite proven treatments. Waiting times are long with poor interface between primary and secondary care. Proper investment needs to be made in the health service to achieve better management of both these conditions and also in public health to reduce the incidence of these diseases for future generations.

GPgeneral practitionerHCPhealthcare professionalHFpEFheart failure with preserved ejection fractionHFrEFheart failure with reduced ejection fractionHFSNheart failure specialist nurseMDTmulti-disciplinary teamNTpro-BNPN-terminal pro b-type natriuretic peptideSOBshortness of breath

HCPs reported high patient group complexity, may indicate the need for an integrated care model which would support both the patients and clinicians. Suggestions included integrating care for specialist input due to the complexity of HFpEF phenotypes and primary care for complex multimorbid illness profiles. HCPs raised the need for the use of non-pharmacologist treatments, prevention and the implementation of any new treatments for HFpEF. There was also an acknowledgement that lack of available treatments, unclear diagnostic criteria, current staff workloads and training and awareness issues in primary care are clear challenges for the implementation of more robust management plans.

### Workshop discussion

**Figure 4:**
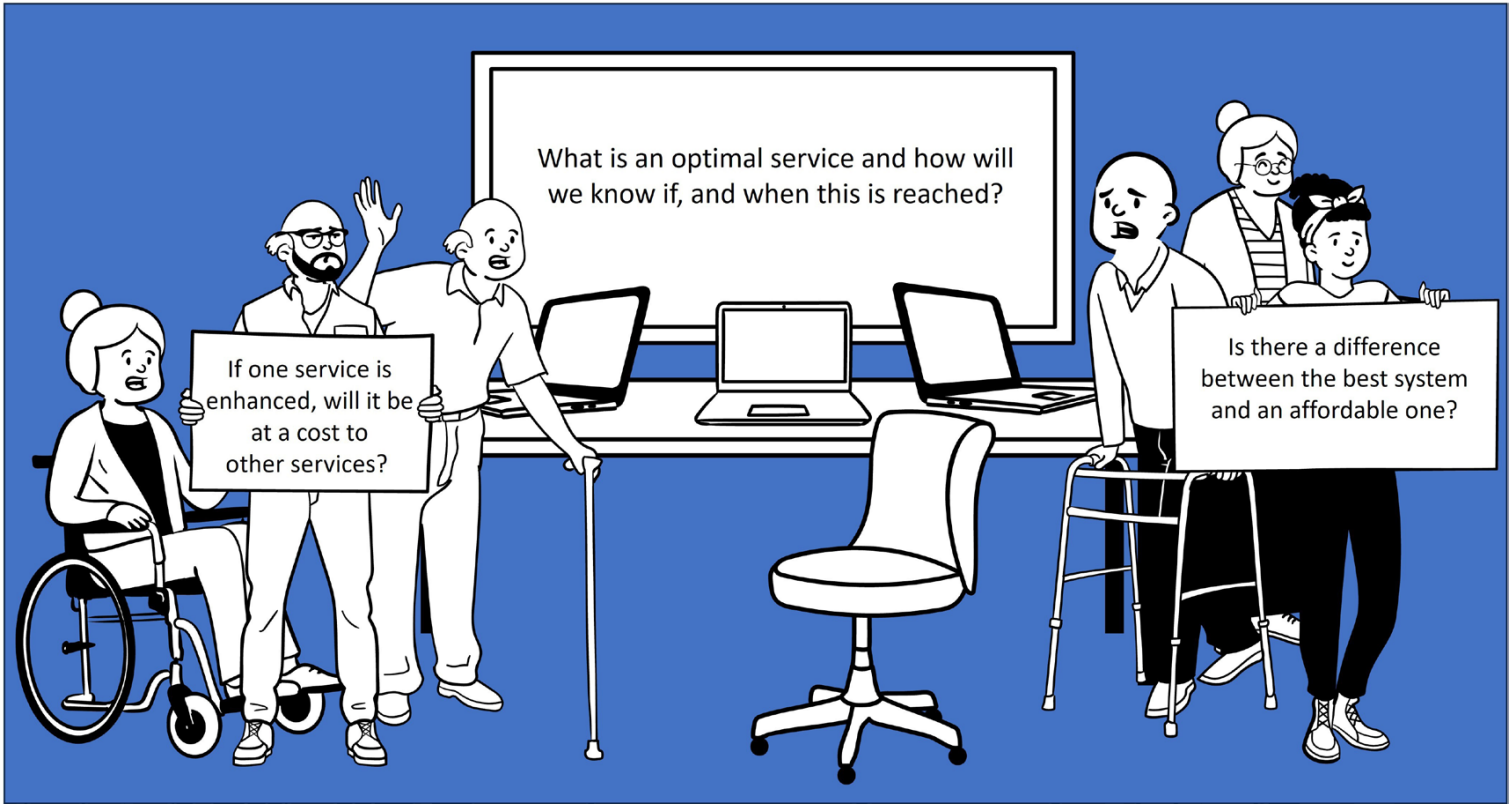
Important questions raised by the workshop group about improving heart failure with preserved ejection fraction management.

Nine HCPs (four HFSN, one GP, three cardiologists and one rehabilitation specialist nurse) and one patient and public representative attended the virtual workshop held in September 2021. The findings of the survey were discussed and attendees confirmed that findings reflected their experiences. Some of the problems highlighted on the post-it notes were delays or long waits for primary care appointments, cardiology referrals and access to echocardiograms. Additional issues around echocardiogram reports (lack of standardisation and missing measurements needed for HFpEF) together with the different NT-pro BNP thresholds (NICE vs ESC) were similar to the survey findings. Workshop participants asked highly relevant questions related to cost and balancing of multiple demands by different groups (see figure 4).

The group discussed potential solutions which were written on the virtual post-it notes which were categorised and provided in table 4.

**Table 4:** Workshop suggested solutions to improving HFpEF diagnosis and management

Category	Potential solutions
Identifying and diagnosing HFpEF	Better codes and improved inclusion on heart failure registers for recalls.
	ICS standard for hf diagnosis subtype always being provided by secondary care.
	EMIS/SYSTM 1 search algorithm (Cuthbert *et al*. 2019), find those on diuretics with touch of breathlessness. Risk assessment, diagnose according to HFA-PEFF algorithm, treat based on patient and clinician joint decision making…
	Clarity of the role of the echo report—offer advice and follow-up guidance like endoscopies.
Education for providers and patients	Upskilling practice nurses to feel confident with heart failure given the supply and demand issue with HFSNs
	1 page primer on how to do a HFpEF QOF review
	Info for patients on the reality of ‘trial and error’ in medication changes.
	Education for GPs
	Patient information leaflets in HF tailored to HFpEF
	A clear guide given to diagnosed patients
	Online education for professionals and patients.
	Educating patients on self-management of fluid overload and cardiovascular risk factors
	Options for access to cardiac rehab—home and communityUtilisation of specialist teams can aid in increasing confidence, myth busting and increasing physical activity/improving outcomes for these patients.
	Better systems for communication between primary and secondary care to aid a more effective and smooth patient journey
	System for rapid advice from heart failure MDT, for example, email/instant message
	Better codes and improved inclusion on heart failure registers for recalls
	Virtual ward rounds where primary and secondary care can attend
	Access to heart failure nurses for people with HFpEF
	Empowering HFSNs as experts to be able to make their own onward referrals if they feel [these are] indicated, for example, to renal [specialist]—streamlining.
	Measure patient centred outcomes plus changes to hospitalisation
	Multiple specialties like geriatrics, Diabetes Specialist Nurses (DSN) [to be involved]
	Integrated care systems
	Medicines management team
	Mental well-being referral and support
Other	Patient support groups to help ongoing management
	Buddy system for new patients
	Voluntary groups/community resources

GPgeneral practitionerHFpEFheart failure with preserved ejection fractionHFrEFheart failure with reduced ejection fractionICSintegrated care systemQOFquality and outcomes framework

## Discussion

### Summary of findings

The results demonstrate that HCPs agreed on improving the diagnosis and management of patients with HFpEF. Consensus was varied in how this goal was to be achieved, possibly related to respondents’ personal and professional context. Most variation was seen in survey questions relating to screening high-risk but asymptomatic individuals, thresholds for NT-proBNP, use of further investigations with uncertain diagnosis and the responsibility for monitoring and managing patients. Free-text comments provided details for participants’ responses and highlighted needs related to management (complex care and a call for integrated care) and barriers (lack of treatments, unclear diagnostic criteria, staff and service capacity) for HFpEF management.

### Diagnosis of HFpEF: importance, tools and documentation

Until recently, there was little incentive to diagnose and manage those with HFpEF. There was a general lack of awareness and interest in HFpEF, and sometimes scepticism about the condition.^6^ As this survey was conducted before the identification of medications to treat HFpEF, the lower percentage of GPs in the survey agreeing that it was very or extremely important to identify type of HF is likely a reflection of previous opinions of HFpEF. The clinical trials of SGLT2 inhibitors in HFpEF^7 8 25^ have encouraged greater interest in HFpEF that has been beneficial over and above the benefit of the drugs themselves.^26^ Our observed general HCP consensus regarding HFpEF diagnostic importance as well as consensus of including type of HF as QOF indicator highlights the shifting perspectives on importance of diagnosing type of HF, prior to the positive pharmacological trials. However, this may also reflect that survey respondents were more likely to be interested in HFpEF, although there were dissenting responses and comments to both questions.

Diagnosis of heart failure can be difficult, as symptoms and signs (shortness of breath, fatigue, ankle oedema and exercise intolerance) are non-specific. Prevalence is higher in older patients who may attribute symptoms to ageing or to other comorbid conditions (as clinicians may do as well).^23 27^,^29^ While consensus regarding assessing symptomatic patients was reached, there was no consensus regarding screening patients at high risk for HFpEF if asymptomatic. The estimated prevalence of all types of heart failure in the general population is widely estimated at 1%–2%, but it is highly likely that many patients with HF—especially HFpEF—remain unidentified.^4 30 31^ A systematic review of echocardiographic screening studies (finding cases of previously unrecognised HF) calculated prevalence in the adult population of 4.2% and 11.8% in people aged 65 and older.^32^ Prevalence of HFpEF was higher than HFrEF, and HFpEF was more likely to be undiagnosed.^29 32 33^ A study in the Netherlands assessed older patients with type 2 diabetes mellitus (T2DM) without previously diagnosed HF.^34^ They found that 28% had cardiologist-confirmed HF, with the majority of the patients with HF (83%) having HFpEF.^35^ Subsequently, the authors argued that screening for HFrEF and HFpEF should be part of the management programme for older patients with T2DM as those found to have HF had significantly worse mortality and hospitalisation rates over the subsequent year.^36 37^ The current increased understanding of HFpEF and development of specific therapies makes the case for screening stronger.

Key diagnostic tools for HF of NT-proBNP and echocardiography divided opinions in our survey. No consensus was reached for thresholds or consideration for NT-proBNP results. But the respondent free-text responses highlighted remaining unclear guidance for thresholds and the consideration of individual patient characteristics. NT-proBNP is the recommended initial test in patients suspected of HF, but the levels at which NT-proBNP is thought to make HF unlikely differ among guidelines (National Institute of Health and Clinical Excellence: 400 pg/mL vs European Society of Cardiology HF125 pg/mL).^9 10 38^ Thresholds for NT-proBNP are affected by patient characteristics/comorbid conditions and drug treatment and debated in HFpEF.^39 40^ An observational study reported that one in five patients with HFpEF may be missed if the higher cut-off value of 400 pg/mL is used.^30^ NT-proBNP is affected by comorbidities (obesity^41^ and atrial fibrillation^41 42^) and certain medications (eg, ACEI^41^). In this survey, GPs, HFSN and cardiologists all called for guidance on evaluating NT-proBNP levels. This aligns with continued calls for improved education and support of clinicians who manage this population.^4043^,^45^ Similarly, there was consensus for improvements to diagnostic echocardiogram reports, but it is important to note that echocardiographic changes in structure and diastolic function indicative of HFpEF may be more challenging to determine and are not always routinely measured.^4^ Raised left ventricular filling pressures in HFpEF may only be evident with exertion or exercise but these tests are seldom performed, and opinions of survey respondents were divided for the need for these tests.

The challenges of diagnosing HFpEF have been recently addressed through risk scores such as the H_2_FPEF score and the Heart Failure Association Pre-test assessment, Echocardiographic and NT-proBNP testing, Functional testing and Final aetiology (HFA-PEFF) score.^46 47^ These scores use a combination of risk factors, NT-proBNP results and echocardiographic parameters to determine the likelihood of HFpEF. A proposed diagnostic algorithm^33^ uses a similar combination of symptoms, NPs (thresholds consistent with ESC guidelines) and echocardiographic parameters at step 1 with the expectation that 80% of patients will be diagnosed at this point. Those with high index of suspicion but normal NT-proBNP levels would be assessed with a risk score at step 2, and only those with intermediate probability of HFpEF would go on to additional testing at step 3.

### Management responsibilities

Although guidance is available for how to diagnose and manage patients with HFpEF, the ability of the healthcare system to deliver the needed services is questionable.^4045 48^,^50^ While consensus was reached (aggregated positive responses) that the diagnosis and initial treatment of HFpEF responsibility should lie within specialist services, management responsibility of this population divided opinions. Consensus was not reached for the subsequent management of patients with HFpEF in primary care, with cardiologists being the main dissenting voice.

Free-text comments highlighted three main barriers to the management of patients with HFpEF: no available treatments, unclear diagnostic criteria and staff and healthcare services capacity. Unclear diagnostic criteria were addressed above and the remaining discussion will focus on the other two barriers.

While the main barrier reported in the free-text comments to HFpEF management was the lack of specific treatment, pharmacological therapy is now available.^7 8 11^ The removal of that barrier does not solve the issues around HFpEF management. Not all services are meeting current demand for HF and delays occur throughout the healthcare system in primary care, access to testing, specialist referral and ongoing community support (Masters J, unpublished data, 2025 and Kwok CS, unpublished data, 2025). Adding in patients with HFpEF places additional burden on struggling services.

In the UK, analyses of HF services in the UK highlight variability across geographical areas, and many services struggle to deliver care with limited resources and long waiting lists, which lead to frustration for GPs and patients (Masters J, unpublished data, 2025 and Kwok CS, unpublished data, 2025). Similar to the results of this consensus study, a review identified that HCPs in primary care highlighted a collaborative approach to overcome the barriers of GP uncertainty and implementing the latest evidence.^44^ Primary care is under similar pressures and limitations in ability to deliver timely care.^48^ Ideally, more resources in the community are needed as well as collaborative models between primary and specialist care that can provide holistic care to the patient with HFpEF who is often complex with multimorbidity and geriatric syndromes. While the results of this survey appear to point towards HFSN management of those with HFpEF, concerns around the capacity of these clinicians were already high when they were only focused on those with HFrEF.^50^

### Limitations and future research

The study was limited by the small sample of providers who completed it, but most regions of the UK were represented. However, the sample was primarily from England, especially the East of England. Participants were likely to be those who had an interest in HFpEF, although some of the comments revealed that not all respondents were knowledgeable on the topic. The representativeness of the sample is limited, probably affected by the pandemic, and likely has a degree of self-selection bias. The three most prevalent HCPs enabled comparisons among HFSN, cardiologists and GPs to highlight areas of consensus and disagreement, but a key voice of practice nurses was limited. Fixed -choice questions limited responses, but the numerous free-text comments highlighted important issues and allowed respondent the freedom to expand on their opinions. Despite the limitation of the survey being conducted prior to recent positive pharmacological trials that demonstrated effectiveness of specific treatments, the survey remains relevant to current challenges. Identified lack of consensus on responsibility for and where patients with HFpEF are diagnosed and managed are more relevant now with available treatment options.

Additional to trials of pharmacological treatment, demonstrating effectiveness in patients with HFpEF, research is needed to develop pathways and models of care of collaborative management of patients with HFpEF and ensure efficient use of resources. Research and testing of innovative models have the potential to overcome entrenched ways of working, break down barriers between specialist services and primary care and improve the diagnosis and management of an underserved group. Recent effective pharmacological therapy are useless, without early identification and appropriate management of patients with HFpEF in an adapted healthcare system.

## Conclusion

This survey demonstrated that across the UK, multiple HCP types agree on the importance of diagnosing HFpEF. At the time of the survey, HCPs identified the main barrier to this was the lack of specific disease-modifying pharmacological treatments. With that barrier now removed, it becomes crucial to resolve the other identified barriers around diagnosis and management of HFpEF. There is a pressing need to ensure the development of appropriate care pathways. HCPs were, and likely remain, divided on where the responsibility for management and diagnosis lies, but were clear on the need for better guidance and care pathways for those with HFpEF. The possibility of developing collaborative and integrated HF care pathways with increased resources for HCPs to care for those with HFpEF was suggested as an initial way to improve the diagnosis and management of this underserved population.

## supplementary material

10.1136/bmjopen-2024-092993 online supplemental file 1

## Data Availability

Data are available upon reasonable request.
