# Supplementary material for: Diagnosing and managing patients with heart failure with preserved ejection fraction: a consensus survey
Source: BMJ Open. 2024 Dec 20;14(12):e092993. doi: 10.1136/bmjopen-2024-092993 (PMC11667415; doi:10.1136/bmjopen-2024-092993)
Supplement: online supplemental file 1 [file bmjopen-14-12-s001.pdf]

CONSENT

Optimise HFpEF Heart failure with preserved ejection fraction (HFpEF): Consensus Methods

You have been invited to take part in this research because you are a member of a professional network who has agreed to cascade this information on our behalf.

What is the purpose of the study?

In this study we want to seek your opinions and ideas on the possible ways to manage HFpEF within the current healthcare system and establish consensus on a way forward that will lead to an improvement in care.

How can I take part?

Taking part involves completing this digital survey. Before taking the survey you will be asked to confirm you are consenting to take part in this research. After completing the consent process you will be asked to provide some basic demographic data and to respond to a series of statements and questions.

Further information and contact details:

If you would like more information please see the participant information sheet [here] or contact the research team at christideatonpa@medschl.cam.ac.uk

I have read the information on the webpage dated [insert], version [insert] for this above study. I have had the opportunity to consider the information, email the project team with questions and have had these answered satisfactorily.

I agree ○

I understand that my participation is voluntary and that I am free to withdraw at any time without giving any reason

I agree ○

I understand that my responses to questions posed by this study will be recorded. I consent for the research team to use this material without my details, possibly including word for word quotes.

I agree ○

I give permission for my responses to questions posed by this study to be stored securely on a password protected computer, in line with the General Data Protection Regulations (May 2018) and Cambridge University Hospitals NHS Foundation Trust local guidelines.

- I agree

QUESTIONS

Please indicate your profession below:

- General practitioner
- Practice Nurse
- Cardiologist
- Heart failure specialist nurse
- Allied health professional
- Echocardiographer
- Other (please state below)

In what region of the country do you practice?

- Greater London
- Southeast
- East of England
- Yorkshire and Humber
- Northeast

- Northwest
- West Midlands
- East Midlands
- Southwest
- Wales
- Northern Ireland
- Scotland
- [ ] Other (please state below)

Please indicate your age range

- 21 - 30
- 31 - 40
- 41 - 50
- 51 - 60
- 61 - 70
- 71 - 80
- 81 - 90
- 91+

Do you identify as:

- Female
- Male
- Non-binary

- ☐ Other
- Prefer not to say

How important do you think it is to diagnose the type of heart failure (i.e. heart failure with reduced ejection fraction [HFrEF] or heart failure with preserved ejection fraction [HFpEF]) a patient has?

- Extremely important
- Very important
- Moderately important
- Slightly important
- Not at all important

Should we assess patients at high-risk of developing HFpEF (i.e. multi-morbid older adults) even if not symptomatic?

- No
- Maybe
- Yes

Should we assess symptomatic patients specifically for HFpEF?

- ☐ No
- ☐ Undecided
- ☐ Yes

P lasma concentration of natriuretic peptides (NPs) can be used as an initial diagnostic test:

The National Institute for Heath and Care Excellence (NICE) recommends a NT-proBNP level > 400 pg/ml
The European Society of Cardiology recommends a NT-proBNP level > 125 pg/ml

Natriuretic peptides are lowered by obesity and active treatment (e.g. diuretics, ACE inhibitors for hypertension) and increased with atrial fibrillation and conditions like diabetes.

What NT-proBNP threshold should be used in symptomatic patients with possible HFpEF? (More than one answer is possible.)

- ☐ > 400 pg/ml
- ☐ > 125 pg/ml
- ☐ Depends on patient characteristics

- ☐ Depends on index of clinical suspicion
- ☐ Better guidance needed

Echocardiogram reports should always state whether there is evidence of heart failure and what type of heart failure this typifies.

Please rate your agreement with the above statement

- ○ Strongly Agree
- ○ Agree
- ○ Somewhat agree
- ○ Neither agree nor disagree
- ○ Somewhat disagree
- ○ Disagree
- ○ Strongly disagree

The Quality Outcomes Framework (QOF) indicators used in general practice for heart failure should be revised to include the type of heart failure documented.

Please rate your level of agreement with this statement.

- ○ Strongly agree
- ○ Agree
- ○ Somewhat agree

- Neither agree nor disagree
- Somewhat disagree
- Disagree
- Strongly disagree

Please provide your rationale for the answer you gave in the question above

A stress or exercise echocardiogram should be performed in patients with suspected HFpEF when the resting transthoracic echocardiogram is inconclusive.

Please rate your level of agreement with this statement

- Strongly agree
- Agree
- Somewhat agree
- Neither agree nor disagree
- Somewhat disagree
- Disagree
- Strongly disagree

A MRI should be performed in patients with suspected HFpEF when the resting transthoracic echocardiogram is inconclusive.

Please rate your level of agreement with this statement

- ○ Strongly agree
- ○ Agree
- ○ Somewhat agree
- ○ Neither agree nor disagree
- ○ Somewhat disagree
- ○ Disagree
- ○ Strongly disagree

Please indicate the response that best reflects your opinion

- ○ Identifying patients with HFpEF is not useful as there is no specific treatment
- ○ Identifying type of heart failure is not useful as treatment will not differ
- ○ It is useful to make a definite diagnosis of the type of heart failure
- ○ Identifying patients with HFpEF is useful as may provide an opportunity to de-prescribe some medications
- ○ Identifying patients with HFpEF is useful as provides an opportunity to focus on comorbidities and lifestyle factors

Diagnosis and an initial treatment plan for patients with HFpEF should be made in specialist services.

Please rate your level of agreement with this statement

- Strongly agree
- Agree
- Somewhat agree
- Neither agree nor disagree
- Somewhat disagree
- Disagree
- Strongly disagree

Management of HFpEF should be in primary care.

Please rate your level of agreement with this statement

- Strongly agree
- Agree
- Somewhat agree
- Neither agree nor disagree
- Somewhat disagree
- Disagree
- Strongly disagree

Heart failure specialist nurses should also manage patients with HFpEF.

Please rate your level of agreement with this statement

- Strongly agree
- Agree
- Somewhat agree
- Neither agree nor disagree
- Somewhat disagree
- Disagree
- Strongly disagree

Practice nurses should be upskilled to manage patients with HFpEF.

Please rate your level of agreement with this statement

- Strongly agree
- Agree
- Somewhat agree
- Neither agree nor disagree
- Somewhat disagree
- Disagree
- Strongly disagree

Please provide any comments or recommendations about the diagnosis and management of patients with HFpEF.

Powered by Qualtrics
