# Supplementary material for: Diagnosing and managing patients with heart failure with preserved ejection fraction: a consensus survey
Source: BMJ Open. 2024 Dec 20;14(12):e092993. doi: 10.1136/bmjopen-2024-092993 (PMC11667415; doi:10.1136/bmjopen-2024-092993)
Supplement: Supplementary file 2 [file bmjopen-2024-092993.reviewer_comments.pdf]

PEER REVIEW HISTORY

BMJ Open publishes all reviews undertaken for accepted manuscripts. Reviewers are asked to complete a checklist review form and are provided with free text boxes to elaborate on their assessment. These free text comments are reproduced below.

ARTICLE DETAILS

Title (Provisional)

Diagnosing and Managing Patients with Heart Failure with Preserved Ejection Fraction: A Consensus Survey

Authors

Austin, Rosalynn; Khair, Eva; Blakeman, Thomas; Hossain, Muhammad Zakir; Sowden, Emma; Chew-Graham, Carolyn; Forsyth, Faye; Deaton, Christi

---

VERSION 1 - REVIEW

**Reviewer**	**1**
**Name**	**Himmelreich, Jelle**
**Affiliation**	**Amsterdam UMC Locatie AMC, General Practice**
**Date**	**13-Sep-2024**
**COI**	

Reviewer comments to:

Diagnosing and Managing Patients with Heart Failure with Preserved Ejection Fraction: A Consensus Survey

(bmjopen-2024-092993)

In reading the manuscript, I had the following remarks/questions (not necessarily in order of importance):

- Strengths of this work are the even distribution of relevant healthcare professionals (primary and secondary care) who responded to the survey; the distinction between important phases in HFpEF patient journey (identification and diagnosis of HFpEF, and responsibilities for management); and the acknowledgement and clarity surrounding a number of limitations including low response rate and the remaining lack of consensus.

- Limitations are that the survey and workshop were held >3 years ago, and were (partly) taken during the COVID epidemic. Moreover, representation at the workshop was not equal between specialties. It is insufficiently clear whether the conclusions from this work are still sufficiently recent and/or can speak for all relevant HCP specialties in the UK.

- In the introductory Strengths/Limitations box, under point two it says "Findings of the survey we confirmed…". Should this be "Findings of the survey were confirmed…"?

- Re "Survey questions were iteratively developed by the research group and the initial survey was piloted with five HCPs leading to refinement." Please clarify how these HCPs were

selected, and what their background/expertise was. Were they related to the authors' research faculties or non-academic professionals?

- Re "Survey questions were iteratively developed by the research group and the initial survey was piloted with five HCPs leading to refinement." Please indicate the outcomes of the pilot/to what extent the survey needed to be changed (e.g. only minor changes in wording, or were entirely new questions added after the pilot, and if so were these questions piloted again to new HCPs)?

- Re "Survey data were collected between December 2020 to March 2021" & "A virtual workshop with healthcare providers and public not involved in the survey was held in September 2021…" - To what extent do the researchers think the answers and response rate were impacted by the COVID epidemic, and would redoing the survey now have influenced the results and/or response rate?

- The results require the number of HCPs to whom the survey was sent out, in order to provide the response rate rather than just the number of responders.

- Re "Sixty-six HCPs completed the survey. Thirty-one percent of respondents came from the East of England region, 43% from seven other regions in England, 12% from Northern Ireland, 7.7% from Wales,1.5% from Scotland, and 9.7% were unknown." (Results) with n=1 from Scotland , the authors might reconsider or rephrase the sentence "geographical representation across the UK was achieved" (Strengths & Limitations box) or might add that the majority were from regions in England

- The authors mention lack of consensus. There is also over-representation of HCPs from regions in England. Was there more consensus among those from England? And should there be a separate analysis/recommendations for England rather that trying to make it work for the entire UK?

- In the Introduction the authors write "two key areas: identification and diagnosis of HFpEF, and responsibilities for management.", while in the Results section the authors write "The survey results were divided into main points for discussion: the diagnosis of HFpEF, inclusion in the QOF framework, and the management of HFpEF" . First: how the survey was analysed seems more fitting in the Methods section than the Results section; second: there is lack of consistence between these statements.

- Although important issues relating to HFpEF are addressed, the paragraphs in the Discussion, e.g. the paragraph starting with "The challenges of diagnosing HFpEF …", do not always have a clear relation with the results from the survey or workshop. This links hould be made more explicitly.

Reviewer **2**

Name **Agvall, Björn**

Affiliation **Halland Regional Hospital, Department of Research and Development**

Date **29-Oct-2024**

COI

This study provides a timely and insightful analysis of the challenges in HFpEF diagnosis and management, seeking to identify areas where perspectives may differ. However, its impact is somewhat constrained by a limited sample size, potential selection bias, and a timing that does not fully reflect recent pharmacological advancements. The following concerns are noted below.

This is an interesting study, and I appreciate the opportunity to read it. As I understand it, the study aimed to assess the level of consensus between primary and secondary care providers on various aspects of HFpEF diagnosis and management. With this perspective, I have some concerns outlined below.

Abstract

In line 31, the sentence begins with numbers (66) and should be spelled out.

The results section primarily consists of commentary on the findings, without providing specific numerical data. To address this, suggest you incorporate relevant numerical results directly in the text.

The objective of the study was to determine the degree of consensus between primary and secondary care providers, but the conclusion does not address this objective. While the challenges of limited resources and difficulty in achieving consensus are acknowledged, they do not directly answer the core question. Is the degree of consensus high or low, or is it considered good or poor, for example?

Introduction

The purpose is unclear and does not fully align with the purpose outlined in the abstract. In the abstract, the purpose is specifically described as determining the degree of consensus, which is clearer than the statement provided in the Introduction section.

Method

How were the participants recruited specifically and what was the selection process? Were they reached through social media and professional networks (which ones), and approximately how many potential individuals could be accessed?

Participation was voluntary and through being interested, which can introduce sampling bias. Those who responded may be more engaged or interested in HFpEF issues than the healthcare providers in general skewing the results toward more informed or motivated participants. Is this taken in consideration?

Setting a consensus threshold at 67% is reasonable but arbitrary and may not adequately capture the complexities of opinions on HFpEF management. Is there a background choosing this threshold?

Results

It is noted that the ages of the participants ranged from 21 to 70 years, and those who are 21 years old may not have had sufficient time to complete medical education or gain experience with HFpEF. Is it known what healthcare provider role this person had?

The results mention the percentage of healthcare providers, but it would be beneficial to also include the actual number. Relying solely on percentages can be misleading.

There appears to be a significant risk that the respondents were primarily individuals who have a personal interest in the topic, raising the question of whether they are representative of the population needed to address the research question. Is this addressed in any way?

In Figure 3. The question that practice nurses should be upskilled to handle HFpEF is a speculative one and has little to do with the research question.

Identifying patients with HFpEF is not useful as there is no specific treatment. Outdated question.

Discussion

The opening is too lengthy and lacks clarity, causing confusion. Recommend condensing to a maximum of 10 lines, focusing on a concise summary of the main findings.

The discussion addresses a wide range of issues with mixed relevance. Suggest refining focus and segmenting content for clarity. Consider separating topics, such as healthcare system
barriers versus diagnostic and management limitations.

The discussion acknowledges the study's small sample size but could further explore how this limitation may impact the generalizability of the findings. Additionally, considering potential biases due to the survey timing (conducted before recent pharmacological trials) would
provide a more transparent perspective on the study's limitations.

In the limitations section, it should be noted that the regional distribution is skewed, with 43% of respondents from England (predominantly the East) and only 1.5% from Scotland. This
imbalance may reduce the representativeness of the findings. Additionally, the small sample size of only 66 HCPs limits the generalizability of the results.

In conclusion, while the importance of diagnosing HFpEF seems consistent across healthcare providers. However, it seems that the management approaches of HFpEF were inconsistent. What identified barriers are important to solve?

Recommend highlighting areas for further research, such as validation of HFpEF risk scores and studies on resource allocation and care model outcomes.

What opportunities exist to shape care programs for HFpEF to achieve greater consensus on diagnosis, and especially in management, where perspectives on HFpEF appear to differ the most?

VERSION 1 - AUTHOR RESPONSE

Reviewer 1 Comments	Response
Limitations are that the survey and workshop were held >3 years ago, and were (partly) taken during the COVID epidemic. Moreover, representation at the workshop was not equal between specialties. It is insufficiently clear whether the conclusions from this work are still sufficiently recent and/or can speak for all relevant HCP specialties in the UK.	We have highlighted this in the limitations and have provided further content in the manuscript and in responses to Reviewer 2 comments. This was a small sample unfortunately, but we believe it reflects important considerations for HFpEF management. The findings are consistent with our previous qualitative work which included interviews with 73 clinicians, and other studies.

- In the introductory Strengths/Limitations box, under point two it says “Findings of the survey we confirmed…”. Should this be “Findings of the survey were confirmed…”?	This has been corrected.
- Re “Survey questions were iteratively developed by the research group and the initial survey was piloted with five HCPs leading to refinement.” Please clarify how these HCPs were selected, and what their background/expertise was. Were they related to the authors’ research faculties or non-academic professionals?	We have added text to provide relevant information. The individuals were known to investigators but not involved in the larger programme of research. Their role was primarily to ensure questions were clear and relevant. All were in clinical practice.
- Re “Survey data were collected between December 2020 to March 2021” & “A virtual workshop with healthcare providers and public not involved in the survey was held in September 2021…” - To what extent do the researchers think the answers and response rate were impacted by the COVID epidemic, and would redoing the survey now have influenced the results and/or response rate?	The participant response rate was likely impacted by COVID (noted in limitations), but not the responses themselves. As we have noted, the recent positive trials have highlighted HFpEF and there is a shift toward more services accepting patients with HFpEF and accepting it as a diagnosis that needs management.
- The results require the number of HCPs to whom the survey was sent out, in order to provide the response rate rather than just the number of responders.	Thank you, but due to the virtual snowball sampling method and the publication of the survey link on social media it is not possible to determine the number of HCPs who may have seen the invite. This was stated in the manuscript. We have updated the manuscript to include the number of survey responders who started but did not complete the survey.
- Re “Sixty-six HCPs completed the survey. Thirty-one percent of respondents came from the East of England region, 43% from seven other regions in England, 12% from Northern Ireland, 7.7% from Wales,1.5% from Scotland, and 9.7% were unknown.” (Results) with n=1 from Scotland , the authors might reconsider or rephrase the sentence “geographical representation across the UK was achieved” (Strengths & Limitations box) or might add that the majority were from regions in England	Thank you this was updated
- The authors mention lack of consensus. There is also over-representation of HCPs from regions in England. Was there more consensus among those from England? And should there be a separate analysis/recommendations for England rather that trying to make it work for the entire UK?	Thank you for this idea, but the response rate was not sufficient to perform this level of analysis.
- In the Introduction the authors write “two key areas: identification and diagnosis of HFpEF, and responsibilities for management.”, while in	Thank you for raising this, we have updated the manuscript to improve consistency.

the Results section the authors write "The survey results were divided into main points for discussion: the diagnosis of HFpEF, inclusion in the QOF framework, and the management of HFpEF" . First: how the survey was analysed seems more fitting in the Methods section than the Results section; second: there is lack of consistence between these statements.	
- Although important issues relating to HFpEF are addressed, the paragraphs in the Discussion, e.g. the paragraph starting with "The challenges of diagnosing HFpEF …", do not always have a clear relation with the results from the survey or workshop. This links should be made more explicitly.	Together with the comments from reviewer two we have reorganized the discussion to address these points.
Reviewer 2 Comments	Response
Abstract	
In line 31, the sentence begins with numbers (66) and should be spelled out.	Thank you, this has been corrected
The results section primarily consists of commentary on the findings, without providing specific numerical data. To address this, suggest you incorporate relevant numerical results directly in the text.	Thank you, we have added some numerical results.
The objective of the study was to determine the degree of consensus between primary and secondary care providers, but the conclusion does not address this objective. While the challenges of limited resources and difficulty in achieving consensus are acknowledged, they do not directly answer the core question. Is the degree of consensus high or low, or is it considered good or poor, for example?	Thank you, we have amended this to make it clearer where there was consensus.
Main Text	
Introduction:<br>The purpose is unclear and does not fully align with the purpose outlined in the abstract. In the abstract, the purpose is specifically described as determining the degree of consensus, which is clearer than the statement provided in the Introduction section.	Thank you, we have corrected to ensure that both are aligned and clear.
Method: How were the participants recruited specifically and what was the selection process? Were they reached through social media and professional networks (which ones), and approximately how many potential individuals could be accessed?	We did state that participants were recruited through professional networks including HCPs who had taken part in the larger programme of research and via social media. We have added more detail to this. WE altered the wording to highlight that there was no selection process;

	and any exclusions were partially completed surveys.
Method: Participation was voluntary and through being interested, which can introduce sampling bias. Those who responded may be more engaged or interested in HFpEF issues than the healthcare providers in general skewing the results toward more informed or motivated participants. Is this taken in consideration?	Thank you, this is a good point. We have had added some content to the limitations regarding this. The individual comments (included in the article) revealed the level of knowledge and interest of respondents.
Method: Setting a consensus threshold at 67% is reasonable but arbitrary and may not adequately capture the complexities of opinions on HFpEF management. Is there a background choosing this threshold?	Thank you, there is no set standard for setting the consensus threshold, but 2/3 is often used according to the "minority obeying majority" principle in a democratic decision, and we have added that reference. The text comments and figures showing breakout of responses help to more adequately capture the complexities of the opinions. We included differences by professional group in the text even when overall consensus is reached.
Results: It is noted that the ages of the participants ranged from 21 to 70 years, and those who are 21 years old may not have had sufficient time to complete medical education or gain experience with HFpEF. Is it known what healthcare provider role this person had?	Thank you for this, as it has highlighted an error we made. We used age categories: 21-30, etc. so that person (a GP) may not have been 21. The highest age range checked was 61 – 70. The text has been altered to clarify these data.
Results: The results mention the percentage of healthcare providers, but it would be beneficial to also include the actual number. Relying solely on percentages can be misleading.<br>There appears to be a significant risk that the respondents were primarily individuals who have a personal interest in the topic, raising the question of whether they are representative of the population needed to address the research question. Is this addressed in any way?	Thank you, we have added in the numbers in partnership with the percentages to increase transparency.<br><br>We have had added some content to the limitations regarding the possibility of self-selection bias.
Results: In Figure 3. The question that practice nurses should be upskilled to handle HFpEF is a speculative one and has little to do with the research question.<br>Identifying patients with HFpEF is not useful as there is no specific treatment. Outdated question.	Thank you, but on the contrary, we think the question of management by upskilled practice nurses or HFSN is very relevant to the consensus issues being explored. There are not enough HFSN currently to manage the needs of all HFrEF patients, much less complex HFpEF patients. Currently HFpEF and much of stable HFrEF is managed in primary care, but primary care is under immense pressure. How we divide and/or collaborate on management of patients is the heart of the matter, and the potential role of PNs is pertinent. We have added further content to highlight these issues in the discussion.

	The question remains relevant. At the time of this survey (prior to recent positive pharmacological trials). HCP clearly identified there was more to the complex issue of HFpEF diagnosis and management than just the existence of pharmaceutical treatments. Recent treatment improvements the debate on diagnostic criteria and management remains even more relevant. Without a clear HFpEF care pathway those effective treatments cannot be offered.<br><br>We have noted this in the content.
Discussion: The opening is too lengthy and lacks clarity, causing confusion. Recommend condensing to a maximum of 10 lines, focusing on a concise summary of the main findings.<br>The discussion addresses a wide range of issues with mixed relevance. Suggest refining focus and segmenting content for clarity. Consider separating topics, such as healthcare system barriers versus diagnostic and management limitations.	Thank you, we have reorganised the discussion section to fit how the results are structured.
The discussion acknowledges the study's small sample size but could further explore how this limitation may impact the generalizability of the findings. Additionally, considering potential biases due to the survey timing (conducted before recent pharmacological trials) would provide a more transparent perspective on the study's limitations.	Thank you, we have noted the timing of the survey and the changes since the positive pharmacological trials.
In the limitations section, it should be noted that the regional distribution is skewed, with 43% of respondents from England (predominantly the East) and only 1.5% from Scotland. This imbalance may reduce the representativeness of the findings. Additionally, the small sample size of only 66 HCPs limits the generalizability of the results.	Thank you, we have noted that most respondents were from the East of England, and the limited generalisability. However, we think the responses are relevant and are consistent with our previous qualitative work and findings from other studies.
In conclusion, while the importance of diagnosing HFpEF seems consistent across healthcare providers. However, it seems that the management approaches of HFpEF were inconsistent. What identified barriers are important to solve?<br>Recommend highlighting areas for further research, such as validation of HFpEF risk scores and studies on resource allocation and care model outcomes.	We have added content around the identified barriers to effective HFpEF management. As well as a section that highlights a few of the needed areas for future research.

What opportunities exist to shape care programs for HFpEF to achieve greater consensus on diagnosis, and especially in management, where perspectives on HFpEF appear to differ the most?	

VERSION 2 - REVIEW

Reviewer 1

Name Himmelreich, Jelle

Affiliation Amsterdam UMC Locatie AMC, General Practice

Date 22-Nov-2024

COI

The questions raised by myself and other reviewers seem to have been addressed appropriately. I have no further comments or questions.

Reviewer 2

Name Agvall, Björn

Affiliation Halland Regional Hospital, Department of Research and Development

Date 23-Nov-2024

COI

I believe the comments have been addressed sufficiently. However, the manuscript includes several typographical errors that need to be corrected. However, the manuscript contains several typographical errors that need to be corrected.
